# Leishmanicidal Action of the Peptides 19-4LF, 19-2.5 and 19-2.5LF Topically Administered on Cutaneous Lesions Caused by *Leishmania major*

**DOI:** 10.3390/pharmaceutics18030332

**Published:** 2026-03-07

**Authors:** Rima El-Dirany, Paolo Ginatta, Celia Fernández-Rubio, Aroia Burguete-Mikeo, Esther Larrea, Guillermo Martinez-de-Tejada, Paul A. Nguewa

**Affiliations:** 1 Department of Microbiology and Parasitology, IdiSNA (Navarra Institute for Health Research), University of Navarra, c/Irunlarrea 1, 31008 Pamplona, Spain; reldirany@unav.es (R.E.-D.); pginatta@alumni.unav.es (P.G.); elarrea@unav.es (E.L.); gmartinez@unav.es (G.M.-d.-T.); 2 Unit of Translational Medicine, IdiSNA (Navarra Institute for Health Research), University of Navarra, c/Irunlarrea 1, 31008 Pamplona, Spain

**Keywords:** neglected disease, cutaneous leishmaniasis, *Leishmania major*, antimicrobial peptides, synergy, in vivo, skin lesion, parasite burden

## Abstract

**Background/Objectives:** Antimicrobial peptides (AMPs) represent a promising class of therapeutics with diverse biological functions, including antibacterial, anti-fungal, anti-parasitic and anti-tumoral activities. Previous works demonstrated the successful repurposing of the two synthetic AMPs 19-2.5 and 19-4LF for cutaneous leishmaniasis, when the compounds were administered in solution on skin lesions caused by *Leishmania major* in a BALB/c mouse model. In this research project, we assessed the activity of 19-4LF, 19-2.5, and their hybrid 19-2.5LF derivative when formulated as a cream for topical administration in the same animal model. **Methods:** The peptides were formulated in DAC cream and applied to the wound of BALB/C mice for 30 days. Lesion progression was monitored using a digital caliper. Parasite burden was measured by qPCR. Parasite viability was assessed using MTT and microscopy imaging assays. **Results:** The three peptides in cream formulation succeeded in reducing the skin lesion. Peptide 19-4LF was the most potent, followed by 19-2.5LF and then 19-2.5. In addition, 19-4LF was able to significantly reduce the parasite burden in the skin lesions of infected mice, as measured by quantifying *L. major Lm18S* ribosomal gene mRNA levels using qPCR. Moreover, when combined, the peptides exhibited synergistic effects on *L. major* promastigotes and significantly reduced the number of amastigotes in infected macrophages. **Conclusions:** These studies support the approach of repurposing these AMPs as antileishmanial drugs and identify 19-4LF as a lead candidate for further studies. While historical barriers to peptide therapeutics included high production costs, recent advancements in biological fermentation and synthesis strategies have significantly improved their economic viability. Furthermore, the use of nanotechnology delivery systems can reduce the required dosage, further making peptide therapy a sustainable option for neglected diseases, including leishmaniasis.

## 1. Introduction

Leishmaniasis is one of the 20 neglected tropical diseases (NTDs) listed by the World Health Organization (WHO). Regrettably, the current drugs available for leishmaniasis treatment are far from optimal, as they face limitations such as toxicity, high cost, and the development of drug resistance. Cutaneous leishmaniasis (CL) is the most common form of the disease and is associated predominantly with morbidity rather than mortality, leading to severe skin ulcers, permanent scarring, and disfigurement, which in turn cause social stigma, psychological distress, and a reduced quality of life for millions of affected individuals. In the case of CL, treatments through topical administrations might be more beneficial than the current intradermal or intravenous injection routes.

To overcome these drawbacks, drug repurposing has emerged as a powerful tool for the discovery of new treatments against neglected diseases including leishmaniasis. This strategy is performed to find new applications for medicines that are already used for other diseases, reducing both the development time and cost. Antimicrobial peptides (AMPs) are promising candidates for drug repurposing. They are an emerging category of therapeutics exerting a wide range of biological activities such as antibacterial, anti-fungal, anti-parasitic and anti-tumoral [1].

Recently, the synthetic anti-lipopolysaccharide peptides (SALPs) Peptide 19-2.5 (Aspidasept^®^, also named 19-2.5 herein) and Peptide 19-4LF (or 19-4LF) have shown leishmanicidal activities, reducing the *Leishmania major* infective burden when administered in solution [2]. Those in vitro and in vivo results suggested that both peptides might be useful for leishmaniasis treatment. On the other hand, 19-2.5LF which is a member of the same family (SALPs) was discovered later, through in vitro assays, to be a promising candidate against leishmaniasis. This work is therefore focused on the leishmanicidal action of the Peptides 19-4LF, 19-2.5 and 19-2.5LF topically administered on skin lesions caused by *Leishmania major*. Herein, to optimize their cutaneous activity, peptides were administered in cream formulation (DAC base cream), a vehicle previously described for topical applications [3]. Interestingly, the peptide 19-4LF achieved a highly significant reduction of lesion size and was associated with a dramatic decrease in parasite burden. Finally, the potential activity of peptide combinations was also assessed. Remarkably, the combinations 19-4LF plus 19-2.5 and 19-4LF plus 19-2.5LF exhibited synergistic leishmanicidal effects on *L. major* promastigotes, as well as a significant reduction of the number of amastigotes in infected macrophages.

## 2. Materials and Methods

### 2.1. Compounds

The peptides included in this study, 19-2.5 (GCKKYRRFRWKFKGKFWFWG), 19-4LF (GKKYRRFRWKFKGKLFLFG), and 19-2.5LF (GCKKYRRFRWKFKGKLFLFG), were synthesized as previously described [3,4,5,6]. Briefly, the solid-phase peptide synthesis was done on a Fmoc-Rink amide resin using an Applied Biosystems 433A synthesizer (Waltham, MA, USA) and the 0.1 mmol FastMoc protocol, including N-terminal Fmoc deprotection. Peptides carried an amidated C terminus. Purity was ≥95% by analytical HPLC and mass spectrometry [3,4,5,6]. The three peptides 19-2.5, 19-4LF and 19-2.5LF were dispersed into a pharmaceutical cream (DAC base cream) for topical application, as previously described [3]. Those formulations were provided by Research Center Borstel (Borstel, Germany). Paromomycin (PM) (Sigma-Adrich, Rome, Italy) was formulated in a water-in-oil cream, as previously described [7].

### 2.2. Animals

Six-week-old male BALB/c mice were obtained from Harlan Interfauna Ibérica S.A. (Barcelona, Spain). Animals were randomly assigned to experimental groups of four and housed in aseptic rooms at the Animal Facility of the University of Navarra. Environmental conditions were strictly controlled, with a 12:12 h light/dark cycle and a constant temperature of 22 °C, and animals had free access to food and water.

All experimental procedures involving animals were reviewed and approved by the Animal Care Ethics Commission of the University of Navarra (approval number 086-20, 30 March 2021). All animal experiments were conducted in accordance with the ARRIVE guidelines [8].

### 2.3. Cells and Culture Conditions

*Leishmania major* (*Lv39c5, Rho/SU/59/P*) promastigotes were kindly provided by Dr. Manuel Soto (Centro de Biología Molecular Severo Ochoa [CSIC-UAM], Madrid, Spain) and were stored at −80 °C in a cryoprotective solution consisting of 90% heat-inactivated fetal bovine serum (HI-FBS) and 10% DMSO. For promastigote experiments, parasites were thawed and cultured at 26 °C in M199 medium (1×; Sigma-Aldrich, St. Louis, MO, USA) supplemented with streptomycin (50 µg/mL), penicillin (50 U/mL), 10% (*v*/*v*) HI-FBS, 25 mM HEPES buffer (pH 7.2), 0.1 mM adenine, 0.0005% (*w*/*v*) hemin, 0.0001% (*w*/*v*) biotin, and 2 mg/mL biopterin.

For amastigote assays, infective parasites were isolated from the spleens of infected BALB/c mice and maintained in Schneider’s *Drosophila medium* (Gibco, Life Technologies Limited, Paisley, UK) supplemented with 10% (*v*/*v*) HI-FBS, penicillin (50 U/mL), and streptomycin (50 µg/mL). Cultures were maintained for a maximum of five passages.

Bone marrow-derived macrophages (BMDMs) used in cytotoxicity and amastigote assays were generated from 6-week-old BALB/c mice, as previously described [7]. Briefly, bone marrow cells were harvested by flushing femurs and tibias with PBS and differentiated for 7 days at 37 °C in a humidified atmosphere containing 5% CO_2_. Cells were cultured in DMEM (1×; Gibco, Life Technologies Limited, Paisley, UK) supplemented with 10% (*v*/*v*) HI-FBS, penicillin (50 U/mL), streptomycin (50 µg/mL), and 20% (*v*/*v*) L929 cell-conditioned medium. L929 cells (ATCC^®^ CCL-1™) were obtained from American Type Culture Collection (ATCC).

The cell lines (*Leishmania major* promastigotes, BMDMs and L929) mentioned in this manuscript had been previously used by our group in biological evaluation assays [2,7,9,10].

### 2.4. L. major Infection and Monitoring of Lesion Progression

Mice were infected by subcutaneous injection of 1 × 10^5^ infective metacyclic promastigotes of *L. major* at the base of the tail. Because lesion development might vary among mice, animals entered the efficacy study at different times once lesions reached a comparable median size. After 6–8 weeks, lesions of measurable size (mean area ≈ 10 mm^2^) had formed, and topical treatments were initiated [7]. The lesion length (L) and width (W) were measured using a digital caliper, and the lesion area was calculated using an ellipse approximation: A = π × (L/2) × (W/2).

In this in vivo pilot study, which prioritized feasibility and animal health status over statistical power, small numbers of animals were included in each experimental group. Five groups were monitored: untreated negative controls, positive controls treated with paromomycin alone, and three additional groups treated with each of the individual peptides. Four treatment groups were then evaluated: paromomycin (PM) (positive control; *n* = 4), 19-2.5 (*n* = 3), 19-4LF (*n* = 4), and 19-2.5LF (*n* = 4). In addition, a negative control group (*n* = 4; vehicle) received vehicle alone (DAC base cream). Treatments were applied topically twice daily for 30 days and consisted of either 50 mg of cream containing 15% PM (1.4 g/kg/day) or 12 mg of cream containing 1% peptide (19-2.5, 19-4LF, or 19-2.5LF; 0.8 mg/kg/day) [11]. Lesion progression was monitored every five days, and body weight was recorded at the end of the treatment period (day 30). Unfortunately, during the study there was a reduction of mice number due to loss of animals and samples failing quality control. Following completion of treatment, animals (PM, *n* = 4; 19-2.5, *n* = 3; 19-4LF, *n* = 4; 19-2.5LF, *n* = 2; vehicle, *n* = 3) remained untreated for three additional days before being sacrificed. Skin lesion samples were aseptically collected, immersed in 0.5 mL of RNAlater solution (Invitrogen, Thermo Fisher Scientific Baltics UAB, Vilnius, Lithuania), and stored at 2–4 °C to prevent RNA degradation.

### 2.5. RNA Extraction

Total RNA from mouse tissue samples was extracted using TRI Reagent (Sigma, St. Louis, MO, USA) according to the manufacturer’s instructions and subsequently treated with DNase (Gibco-BRL) for 20 min at 37 °C, quantified in a NanoPhotometer^®^ NP80 (Implen GmbH, München, Germany) and stored at −80 °C until later use.

### 2.6. Parasite Burden Evaluation from Infected Mice

The parasite load in different mouse tissues was determined by quantifying mRNA levels of the 18S ribosomal gene (*Lm18S*) from *L. major*. For that, one µg of DNase-treated total RNA from each sample was used for reverse transcription with M-MLV reverse transcriptase (Invitrogen) at 37 °C for 1 h. The obtained cDNA was used for the real-time qPCR assays using the CFX Connect real-time PCR detection system (Bio-Rad Laboratories (Singapore) Pte Ltd., Singapore), 96-well plates and IQ SYBR Green supermix (both from Bio-Rad Laboratories, Hercules, CA, USA), as previously described [12]. The sequences of the specific primers for *Lm18S* were as follows: forward primer (Fw) 5′-CCAAAGTGTGGAGATCGAAG-3′ and reverse primer (Rv) 5′-GGCCGGTAAAGGCCGAATAG-3′. Mouse *β-actin* (Fw: 5’- CGCGTCCACCCGCGAG-3’ and Rv: 5’- CCTGGTGCCTAGGGCG-3’) was used as the reference gene to normalize *Lm18S* gene expression [2,7,12,13]. All qPCRs were performed at a 60 °C primer annealing temperature. To monitor the specificity, the final PCR products were analyzed by melting curves and electrophoresis gel. The PCR efficiency was 95% and 102% for each pair of primers, respectively. The samples were tested in duplicate. Controls as no-template and qPCR without reverse transcription were also performed. The amount of each transcript was expressed relative to the reference gene mouse *β-actin* as 2^∆cq^, where cq represents the difference in quantification cycle between the control *β-actin* and *Lm18S* gene. Then, the data were normalized to the control group.

### 2.7. Sequence Alignments

Multiple sequence alignments were generated using Clustal Omega version 1.2.4, accessed through the European Molecular Biology Laboratory–European Bioinformatics Institute (EMBL-EBI) web server (https://www.ebi.ac.uk/Tools/msa/clustalo/) accessed on 5 September 2023 [14].

### 2.8. Promastigote Viability

Log-phase promastigotes (2.5 × 10^6^ cells/mL) were seeded into 96-well plates at a volume of 100 µL per well. Test compounds diluted in M199 medium were added (100 µL per well), and cultures were incubated at 26 °C. The final concentrations were 5, 2.5 and 1.25 µM for all peptides, alone and in combination. After 48 h, cell viability was assessed using the MTT assay (3-[4,5-dimethylthiazol-2-yl]-2,5-diphenyltetrazolium bromide; Sigma, St. Louis, MO, USA) [9]. MTT was added to a final concentration of 0.45 mg/mL, followed by incubation for 4 h under the same conditions. Formazan crystals were dissolved by adding 80 µL of DMSO, and absorbance was measured at 540 nm using a Multiskan SkyHigh plate reader (Thermo Fisher Scientific, Life Technologies Holdings Pte Ltd., Singapore). The percentage of growth inhibition (*PGI*) was calculated as: *PGI* = (1 − (*OD_sample_*/*OD_control_*)) × 100. Two independent experiments (biological replicates) were performed for all biological assays, each one normally with four technical replicates. Untreated parasites were used as negative controls.

### 2.9. Amastigote Viability (Intracellular Leishmanicidal Activity)

BMDMs (Bone marrow-derived macrophages) were seeded onto 8-well chamber slides (Lab-TekTM; BD Biosciences, East Rutherford, NJ, USA) at a density of 5 × 10^4^ cells per well in DMEM and allowed to adhere overnight at 37 °C in 5% CO_2_. Infective metacyclic promastigotes were isolated by peanut agglutinin (PNA) selection [15] and added at a parasite-to-macrophage ratio of 10:1. After 24 h of incubation, non-internalized parasites were removed by PBS washing.

Peptides 19-4LF and 19-2.5 were prepared in DMEM at final concentrations of 5 µM and 10 µM, respectively, and 200 µL of the mixture was added to each chamber. After 48 h, cells were washed with PBS, fixed with ice-cold methanol for 5 min, and stained with Giemsa (1:20 dilution in water) for 20 min. Untreated cells were used as negative controls.

Bright-field images were captured at 40× magnification and processed using Fiji (ImageJ2, v2.16.0/1.54p) to remove the background and adjust brightness. Images were segmented and digitally rescaled to approximate 100× magnification using standard Python (v3.10.13)-based image processing workflows. Quantification was carried out using a YOLO v8 model trained and validated by Sorrilha-Rodrigues et al. (2024), executed with the previously described script [16] available via Google Colab (https://drive.google.com/drive/folders/1X4IYxIZZ1TXysNRY1vS4zeIt30cdwuwr?usp=sharing, accessed 22 July 2025). Parasite burden was expressed as the total number of intracellular amastigotes per 100 macrophages, with more than 1000 macrophages analyzed per condition.

### 2.10. Synergy Score Model

Bliss synergy scores were calculated using the Synergy Finder Plus web application (version R-3.10.3) [17]. The Bliss independence model assumes that combined drug effects occur independently. Deviations from this expected behavior are interpreted as synergistic or antagonistic interactions, indicating non-independent drug action [18,19,20]. Heatmaps generated by the application were downloaded for analysis.

### 2.11. Statistical Analyses

Statistical analyses were conducted using GraphPad Prism software (version 9.0.1). Results are presented as mean ± standard deviation (SD). Comparisons between two groups were performed using a two-tailed unpaired *t*-test. Statistical significance was defined as ** *p* < 0.001, * *p* < 0.01, and *p* < 0.05. Confidence intervals for synergy scores were obtained directly from the Synergy Finder Plus web application (version R-3.10.3) [17].

## 3. Results

### 3.1. Homology and In Vitro Leishmanicidal Activity of Peptides 19-2.5, 19-2.5LF and 19-4LF

**Figure 1:**
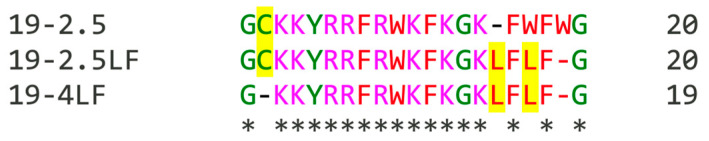
Alignments of the amino acid sequences of 19-2.5, 19-2.5LF and 19-4LF. An asterisk (*) means positions with a single, fully conserved residue. The yellow background highlights the partially conserved residues between the peptides.

The alignments carried out using the Clustal Omega program revealed that 19-2.5, 19-2.5LF and 19-4LF share high similarity in their sequences, with slight differences in N-terminal and C-terminal residues (Figure 1). Interestingly, 19-2.5 LF is somewhat a hybrid of 19-2.5 and 19-4LF, with GC residues at the N-terminus, as in 19-2.5, and LFLF residues at the C-terminus, as in 19-4LF (Figure 1). Besides 19-2.5 and 19-4LF, 19-2.5LF was also included in the analysis, as it had shown promising activity in previous studies [2].

### 3.2. Peptides Topically Administered in Cream Formulation Reduce Lesion Size of L. major-Infected BALB/c Mice

**Figure 2:**
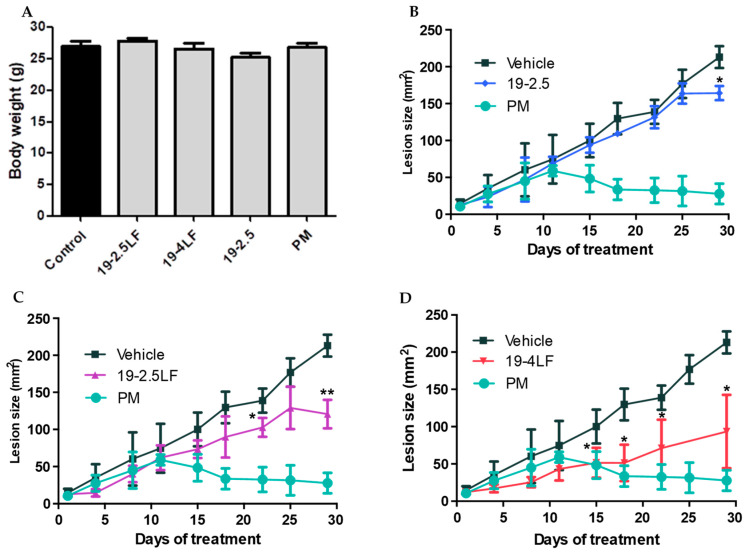
In vivo studies. (**A**) Weight of *L. major*-infected BALB/c mice after 30 days of treatment with a topical cream (vehicle) containing 1% peptide 19-2.5LF, 19-4LF or 19-2.5, compared to infected untreated (i.e., receiving the vehicle alone) mice. (**B**–**D**): Lesion size progression of *L. major*-infected BALB/c mice during 30 days of treatment with topical cream (vehicle) containing 1% peptide 19-2.5 (*n* = 3) (**B**), 19-2.5LF (*n* = 4) (**C**) or 19-4LF (*n* = 4) (**D**), compared to infected mice treated with the vehicle alone (control). The mice treated with Paromomycin (PM) were used as positive control. Results are expressed as mean ± SD. Statistically significant changes compared to the control are indicated (* *p* < 0.05; ** *p* < 0.01).

To evaluate their in vivo efficacy, the peptides were administered topically. For the cream formulations, vehicle cream was applied alone (control) and in combination with 19-2.5LF, 19-4LF or 19-2.5. A cream of Paromomycin, already used in clinic for lesion application [7,21], was administered as positive control. In infected BALB/c mice and regardless of the treatment, the lesion size progressively increased in all the animals until reaching an average area of 50–60 mm^2^ on day 12. At later timepoints, however, this parameter differed markedly depending on the treatment. Interestingly, no loss of weight was observed in any treatment group (including that receiving Paromomycin cream) in comparison to untreated animals (control). In agreement with previous studies [22], this result indicates that mice maintained a good health status during the treatment (Figure 2A).

On the other hand, mice treated with the vehicle (i.e., cream alone; control) showed a continuous increase in lesion size (Figure 2B–D). However, when animals received the cream containing peptides, the lesion size decreased significantly, although the time of this effect varied depending on the specific peptide. In fact, 19-2.5 was the least effective, as a reduction in lesion size was observed only on the last day of treatment (* *p* < 0.05, *n* = 3) (Figure 2B). Peptide 19-2.5LF was more effective than 19-2.5, being able to reduce the skin lesion size from day 18 (Figure 2C) until the end of treatment (day 30, ** *p* < 0.01, *n* = 2). This result will be further discussed. Finally, 19-4LF was the most effective treatment, reducing the skin lesion size from day 15 until the end (* *p* < 0.05, *n* = 4), with reductions of a similar magnitude as those achieved with the reference treatment (i.e., Paromomycin; Figure 2D).

### 3.3. 19-4LF Significantly Reduces the Parasite Burden In Vivo When Topically Administered

**Figure 3:**
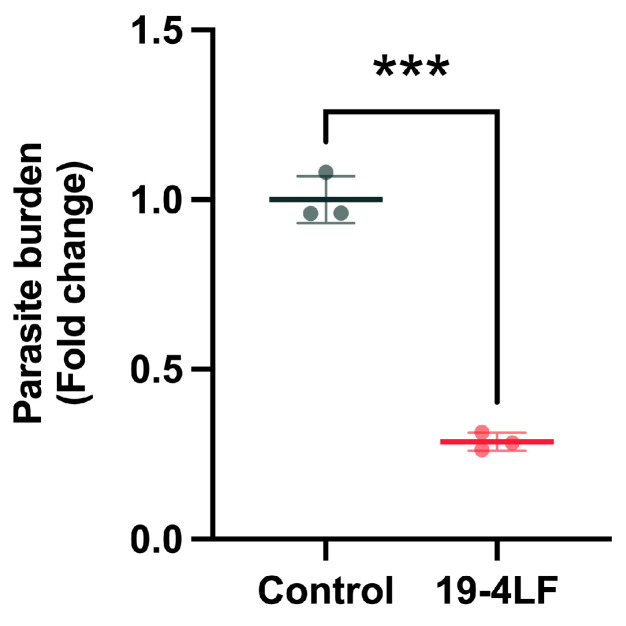
Effect of peptide 19-4 LF in cream formulation on parasite burden in the skin lesions of *L. major*-infected mice after 30 days of treatment compared to controls (mice treated with the vehicle cream alone). The parasite burden was evaluated by quantifying the *Lm18S* gene expression levels using qPCR. Bars represent mean ± SD. Significant differences are indicated (*** *p* < 0.001) compared with the control.

To evaluate the leishmanicidal activity of the treatments, the parasite burden was assessed on day 30 by extracting total RNA from skin lesion tissues and quantifying *Lm18S* mRNA levels using qPCR. In mice treated with PM, there was no expression of the parasite gene *Lm18s*, which indicated that PM caused the death of almost all the parasites in the skin lesion of infected mice. Similarly, 19-4LF dramatically decreased the parasite burden in skin lesions compared to the control (samples treated with the vehicle cream alone) (***; *p* < 0.001) (Figure 3). Such a reduction was associated with the observed reduction of the lesions size by 19-4LF (Figure 2D). Therefore, these results confirm the relevant activity of the lead 19-4LF.

### 3.4. The Combinations 19-4LF Plus 19-2.5 or 19-2.5LF Dramatically Inhibit the Growth of Leishmania Promastigotes

**Figure 4:**
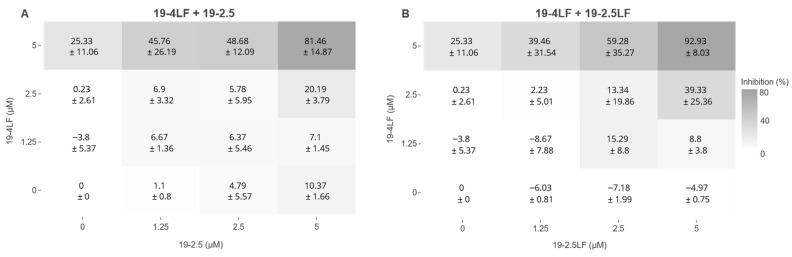
Percentage of growth inhibition of *Leishmania major* promastigotes exposed to peptides alone and in pairwise combinations. The parasite growth inhibition was measured for the 19-4LF + 19-25 (**A**) and the 19-4LF + 19-25LF (**B**) peptides combinations. The corresponding data are represented as the mean ± SD from two independent biological samples. The intensity of gray corresponds to the percentage of inhibition.

The leishmanicidal activity of single peptides (19-4LF and 19-2.5) previously reported in in vitro assays [2] was further demonstrated by the in vivo experiments presented here (Figure 2 and Figure 3). Next, we analyzed the potential synergistic activity of the three peptides under study in pairwise (double) combinations. Figure 4 shows the percentage of growth inhibition of *L. major* promastigotes after exposure to the indicated peptide combinations. In both cases (19-4LF plus 19-2.5 (Figure 4A) and 19-4LF plus 19-2.5LF (Figure 4B)), concentration-dependent increases in drug activities were observed. Notably, at the concentrations tested (0–5 µM), each peptide alone displayed very low or even null leishmanicidal activity (Figure 4). In fact, the mean rates of parasite growth inhibition were <30% in the single-peptide treatments. In marked contrast, peptide combinations, particularly at 5 µM, dramatically inhibited promastigote growth, with mean rates > 80% (Figure 4).

### 3.5. 19-4LF Exhibits Synergistic Interactions with 19-2.5 or 19-2.5LF

**Figure 5:**
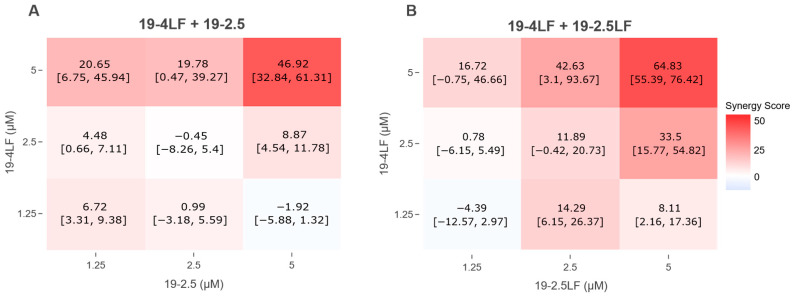
Synergy score heatmaps. The heatmaps show the concentration-specific synergy scores (and their corresponding 95% confidence interval) of the combination of 19-4LF with (**A**) 19-2.5 and (**B**) 19-2.5LF. Positive (red) scores indicate synergistic effects; negative scores indicate antagonistic effects. Experiments were conducted in duplicate (*n* = 2).

To determine whether these effects were simply additive or the result of true synergistic interactions, peptide combinations were analyzed using the Bliss method [19,23]. This mathematical model yields synergy scores, in which positive values indicate synergy, scores around zero denote additive effects, and negative values reflect antagonism. The mean synergy scores (with their respective 95% confidence intervals) are shown in Figure 5. As expected, the most potent synergistic interactions occurred at the 5 µM + 5 µM combinations of 19-4LF + 19-2.5 and 19-4LF + 19-2.5LF, with synergy scores of 46.92 (95% CI: [32.84, 61.31]) and 64.83 (95% CI: [55.39, 76.42]), respectively.

### 3.6. The Combination 19-4LF Plus 19-2.5 Significantly Reduces L. major Amastigote Burden in Murine Macrophages

**Figure 6:**
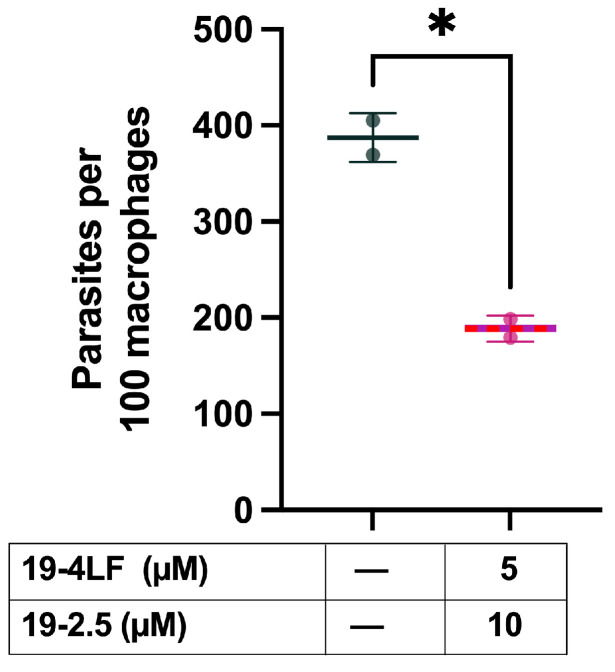
Burden of *L. major* amastigotes after 48h treatment with drug combination. Mean (± SD) parasite burden per 100 infected macrophages treated with 19-4LF (5 µM) + 19-2.5 (10 µM) (indicated in red) compared to controls (untreated infected macrophages; indicated in black). Data represents the mean ± SD of two independent experiments. Significant differences are indicated (* *p* < 0.05).

To validate the synergistic effects of the combinations as candidates for leishmaniasis therapy, we evaluated the ability of 19-4LF in combination with 19-2.5 to reduce the *L. major* amastigote burden in murine macrophages. Interestingly, the combination regime 5 µM 19-4LF plus 10 µM 19-2.5 caused a significant drop in the number of amastigotes in *L. major*-infected macrophages (Figure 6). Specifically, the number of amastigotes per 100 macrophages was reduced almost by 50%, from 387.2 ± 25.4 in the untreated control to 188.9 ± 13.56 when treated with the peptide combination.

## 4. Discussion and Conclusions

Leishmaniasis is one of the most important neglected infectious diseases worldwide, according to the World Health Organization. Even though it is a global and impactful pathology, its elimination remains challenging [24], and pending goals include drug resistance and toxicity, treatment duration and high cost [25,26]. In addition, canonical drug development is time-consuming, and bringing a new drug to market may take 10–15 years [27]. Currently, drug repurposing is an emerging strategy that seeks to discover new therapeutic applications for already available drugs. In the case of *Leishmania* and other trypanosomatids, we and others have demonstrated that antimicrobial peptides, originally designed to target bacteria, can be successfully repurposed for use against these parasites [28]. For instance, we showed that the antibacterial peptides 19-2.5 and 19-4LF exhibited significant antileishmanial activities [2]. Additionally, previous in vivo studies with 19-2.5 and 19-4LF in a BALB/c mouse model of cutaneous leishmaniasis demonstrated reductions in the parasite burden in the skin lesions and the spleen, as well as modulation of cytokines involved in the immune response [2]. To explore potential leishmanicidal effects, in the present work, we decided to test 19-2.5LF, which combines a high sequence similarity to 19-2.5 at its N-terminal end and to 19-4LF at its C-terminus.

On the other hand, peptides are known to be unstable when administered orally or systemically, as peptidases and proteases present in the plasma, liver and kidneys may rapidly degrade them, leading to their elimination via the urine [29]. To reduce these detrimental effects, the peptides were formulated in a pharmaceutical cream (DAC base cream), a vehicle that has been shown to minimize peptide degradation by skin proteases [3]. Here, we show that the three peptides in cream formulation reduced the skin lesion size caused by *L. major* in BALB/c mice, being 19-4LF the most potent, followed by 19-2.5LF and 19-2.5. Furthermore, 19-4LF was able to significantly reduce the parasite burden in the skin lesion of BALB/c mice. Interestingly, these results are consistent with our previous observations in the same animal model using peptides in solution. In those experiments, 19-4LF displayed higher potency than 19-2.5, as assessed by its ability to lower the parasite burden in both the skin lesion and the spleen of infected animals [2]. Taken together, these findings point at 19-4LF as a promising candidate for the treatment of cutaneous leishmaniasis. Despite the historical perception of peptides as high-cost therapeutics, recent technological shifts are significantly improving their economic feasibility for neglected diseases. Advances in recombinant production and large-scale manufacturing processes [30,31] have paved the way for more affordable synthesis. Furthermore, the development of green chemistry protocols, such as ‘no-wash’ strategies and faster manufacturing methods, aims to reduce reagent waste and production time, potentially lowering the market price of peptide-based medicines [32]. The integration of computational design also allows for the identification of more stable and potent analogs, which could reduce the required therapeutic dose and overall treatment costs [33]. Consequently, 19-4LF remains a viable candidate for further development, regardless of current economic challenges. Moreover, while the observed high standard deviation and small sample size in our in vivo model are acknowledged limitations, the consistent trend toward clinical improvement provides a strong proof of concept. These preliminary results justify future larger-scale studies to confirm the robustness of the 19-4LF-containing cream and to optimize its delivery consistency.

In addition, our results also confirmed the antileishmanial potential of 19-2.5LF. Since this peptide shares the same C-terminal sequence (i.e., LFLF) with 19-4LF, and both compounds are more potent than 19-2.5, it is likely that this motif confers enhanced anti-leishmanial activity. Notably, this trend is also evident in antibacterial assays, as 19-4LF was reported to exhibit 4–16 times higher antibacterial activity than 19-2.5 [34]. Conversely, the presence in 19-2.5 of the FWFW domain, composed of bulky aromatic amino acids, is likely to hinder peptide interaction with potential molecular targets. Also, this domain may be responsible for the reduction in its intrinsic solubility (Supplementary Figure S1) when compared to the other two. This may account for previous findings in which 19-2.5 appeared predominantly as a dimer following extraction from DAC cream, whereas 19-4LF remains monomeric [3]. This self-association may reduce drug availability and therefore decrease its activity.

In this study, individual drug administration was associated with significant reductions in lesion size and parasite burden in in vivo models. Regarding peptides, it is important to note their natural tendency to perform better in combination rather than as single agents, which can be advantageous for therapy. Indeed, evidence suggests that certain peptides interact synergistically with each other and with other antimicrobials in nature [35]. Consistent with this, our in vitro assays showed that 19-2.5 or 19-2.5LF combined with 19-4LF achieved greater inhibition of *Leishmania* promastigote growth compared to single-peptide treatments. Similarly, combination of 19-4LF with 19-2.5 (the most extensively characterized member of this peptide family) served as a benchmark to test whether the combinations’ effectiveness translates to the intracellular stage. Previous studies have proposed that genetic selection may favor organisms producing AMP cocktails as a cost-efficient strategy to reduce microbial loads in hosts [36]. Therefore, elucidating the mechanisms underlying synergistic activity when AMPs are combined with other antimicrobials remains a major challenge [37].

Using a known cream formulation containing synthetic antimicrobial peptides, this work proposes a potential topical candidate for cutaneous leishmaniasis with moderate in vivo efficacy. This proof-of-concept study evaluated relevant factors, such as the topical route of administration and the ability of the formulation to protect peptides from degradation, a phenomenon described in previous studies [3]. When formulated in a cream and administrated topically as single agents, 19-4LF, 19-2.5, and 19-2.5LF were associated with the reduction of the skin lesion size, being 19-4LF the most potent, followed by 19-2.5LF and finally 19-2.5. Importantly, we demonstrated that combinations of these peptides (i.e., 19-4LF plus 19-2.5 and 19-4LF plus 19-2.5LF) exhibited synergistic leishmanicidal activity in vitro. In spite of the significant findings reported, this study has limitations that warrant consideration. The sample sizes were small and uneven (*n* = 3–4 per group), which may reduce the overall statistical power. Specifically, the reduction in animal numbers (in the 19-2.5LF and control groups) was due to loss of animals and samples failing quality control, resulting in an unbalanced design. While minimizing animal usage was prioritized in this preclinical proof-of-concept study, and the observed effects reached statistical significance, these results should be interpreted as preliminary. Future studies with larger cohorts are necessary to confirm these trends and ensure broader generalizability of the observed biological phenomena.

## Data Availability

The data presented in this study are available on request from the corresponding author due to confidentiality agreements.

## Supplementary Materials

The following supporting information can be downloaded at: https://www.mdpi.com/article/10.3390/pharmaceutics18030332/s1, Figure S1. Predicted aqueous solubility for 19-2.5, 19-2.5LF and 19-4LF. References [38,39,40,41] are cited in the supplementary materials.

## Abbreviations

The following abbreviations were used in this manuscript:

CL	Cutaneous leishmaniasis
AMPs	Antimicrobial peptides
SALPs	Synthetic anti-lipopolysaccharide peptides
MTT	3-[4,5-dimethylthiazol-2-yl]-2,5-diphenyltetrazolium bromide
DMSO	Dimethyl sulfoxide
PM	Paromomycin
