# Supplementary material for: Leishmanicidal Action of the Peptides 19-4LF, 19-2.5 and 19-2.5LF Topically Administered on Cutaneous Lesions Caused by Leishmania major
Source: Pharmaceutics. 2026 Mar 7;18(3):332. doi: 10.3390/pharmaceutics18030332 (PMC13028876; doi:10.3390/pharmaceutics18030332)
Supplement: Supplementary file 1 [file pharmaceutics-18-00332-s001.zip › pharmaceutics-4093258-supplementary.pdf]

Article

Leishmanicidal action of the Peptides 19-4LF, 19-2.5 and 19-2.5LF topically administered on cutaneous lesions caused by *Leishmania major*

Rima El-Dirany [1], Paolo Ginatta [1], Celia Fernández-Rubio [1], Aroia Burguete-Mikeo [1], Esther Larrea [1,2], Guillermo Martinez-de-Tejada [1] and Paul A. Nguewa [1,*]

[1] Department of Microbiology and Parasitology, IdiSNA (Navarra Institute for Health Research), University of Navarra, c/Irunlarrea 1, 31008 Pamplona, Navarra, Spain; reldirany@unav.es (R.E.-D.); pginatta@alumni.unav.es (P.G.); elarrea@unav.es (E.L); gmartinez@unav.es (G.M.-d.-T.).
[2] Unit of Translational Medicine, IdiSNA (Navarra Institute for Health Research), University of Navarra, c/Irunlarrea 1, 31008 Pamplona, Navarra, Spain;
* Correspondence: panguewa@unav.es

Aqueous solubility for 19-2.5, 19-2.5LF and 19-4LF

As shown below, a brief *in-silico* comparison of predicted aqueous solubility for the three peptides (e.g., pI/charge-based and sequence-based predictors) suggests that 19-2.5, 19-2.5LF and 19-4LF may exhibit good water solubility. In fact, solubility can be analyzed (**Fig. S1**) by useful tools such as Innovagen PepCalc [38, 39], CamSol-PTM [40] and the MahLool solubility prediction tool [41], available at https://pepcalc.com, https://www-cohsoftware.ch.cam.ac.uk/index.php, and www.peptide.bio, respectively.

The three peptides are predicted to be water-soluble by charge/pI-based estimation (PepCalc) and by a learned solubility classifier (MahLooL; 83–91% probability). Through additional studies, CamSol-PTM intrinsic solubility at pH 7.4 differentiates the sequences: 19-2.5 shows a moderately reduced intrinsic solubility score (−0.74) compared to 19-2.5LF (+0.21) and 19-4LF (+0.26). In CamSol-style scoring, negative values indicate solubility-limiting sequence features, while values near 0 indicate negligible net effect, and scores below −1 are commonly interpreted as poor solubility signals.

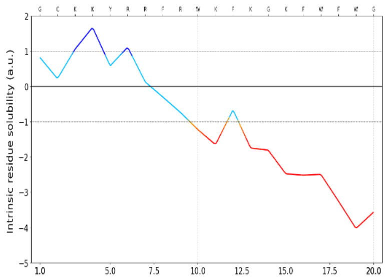

19-2.5

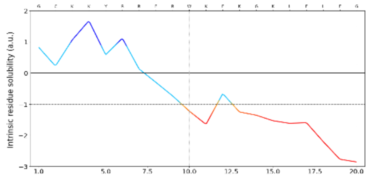

19-2.5LF

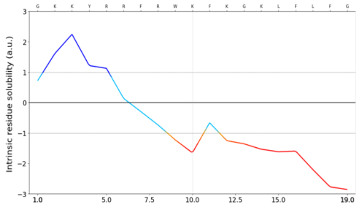

19-4LF

Peptide	CamSol-PTM intrinsic solubility (pH 7.4)	Innovagen PepCalc pI	Net charge (pH 7)	PepCalc estimated solubility	MahLooL / peptide.bio classification	MahLooL probability
19-2.5	−0.737534	11.54	7.9	Good water solubility	Soluble	91%
19-2.5LF	+0.207398	11.54	7.9	Good water solubility	Likely soluble	83%
19-4LF	+0.257793	11.96	8.0	Good water solubility	Likely soluble	83%

Figure S1. Predicted aqueous solubility for 19-2.5, 19-2.5LF and 19-4LF.

References

38. Duan, X.; Gao, X.; Leng, Y.; Xia, B.; Bu, G.; Yang, C.; Zhu, T.; Chen, F. Separation, Identification and Chelation Mechanism of Novel Zinc-Chelating Peptides from Soybean Protein Enzymatic Hydrolysate. *LWT* **2025**, *233*, 118518, doi:10.1016/j.lwt.2025.118518.
39. Wu, N.; Yang, Z.; Wu, C.; Chen, Y.; Chan, Z.; Zeng, R. Purification and Characterization of Anti-Inflammatory Peptide Fractions from Enzymatic Hydrolysate of Abalone Viscera. *Foods* **2025**, *14*, 3811, doi:10.3390/foods14223811.
40. Oeller, M.; Kang, R.J.D.; Bolt, H.L.; Gomes Dos Santos, A.L.; Weinmann, A.L.; Nikitidis, A.; Zlatoidsky, P.; Su, W.; Czechtizky, W.; De Maria, L.; et al. Sequence-Based Prediction of the Intrinsic Solubility of Peptides Containing Non-Natural Amino Acids. *Nat Commun* **2023**, *14*, 7475, doi:10.1038/s41467-023-42940-w.
41. Ansari, M.; White, A.D. Serverless Prediction of Peptide Properties with Recurrent Neural Networks. *J. Chem. Inf. Model.* **2023**, *63*, 2546–2553, doi:10.1021/acs.jcim.2c01317.
